# A Case Report of Ulcerative Colitis Induced by Therapy of Colorectal Carcinoma

**DOI:** 10.5005/jp-journals-10018-1148

**Published:** 2016-07-09

**Authors:** Tuncer Temel, Safak Meric Ozgenel, Funda Canaz, Deniz Arik, Salih Tokmak, Aysegul Harmanci Ozakyol

**Affiliations:** 1 Department of Gastroenterology, Eskisehir Osmangazi University, Eskisehir, Turkey; 2 Department of Pathology, Eskisehir Osmangazi University, Eskisehir, Turkey

**Keywords:** Capecitabine, Colectomy, Colorectal carcinoma, Ulcerative colitis.

## Abstract

Although patients with ulcerative colitis have an increased risk for colon cancer which is associated with disease activity, location of involvement or the accompanying primary sclerosing cholangitis, ulcerative colitis induced by resections for colorectal carcinoma or chemotherapy drugs are very rare as case presentations in the literature.

Fifty-nine year-old female patient with the diagnosis of sigmoid colon carcinoma have been developed ulcerative colitis 2 months after low anterior resection and oral capecitabine treatment.

Development of colitis after colon cancer may be associated with some causes as mutual genetic factors that take part at the pathophysiological mechanisms liable from occurrence of ulcerative colitis and colorectal carcinoma, chemotherapy agents, perioperative stress and underlying silent ulcerative colitis. It is unclear which role is certain. Increasing reports like this case will be useful in resolving this issue.

**How to cite this article:**

Temel T, Ozgenel SM, Canaz F, Arik D, Tokmak S, Ozakyol AH. A Case Report of Ulcerative Colitis Induced by Therapy of Colorectal Carcinoma. Euroasian J Hepato-Gastroenterol 2015;5(2):115-117.

## INTRODUCTION

Although patients with ulcerative colitis have an increased risk for colon cancer,^1^ ulcerative colitis induced by resections for colorectal carcinoma or chemotherapy is very rare. Here, we presented a case of ulcerative colitis case that occurs after resection for colorectal carcinoma and chemotherapy administration.

## CASE REPORT

**Fig. 1:**
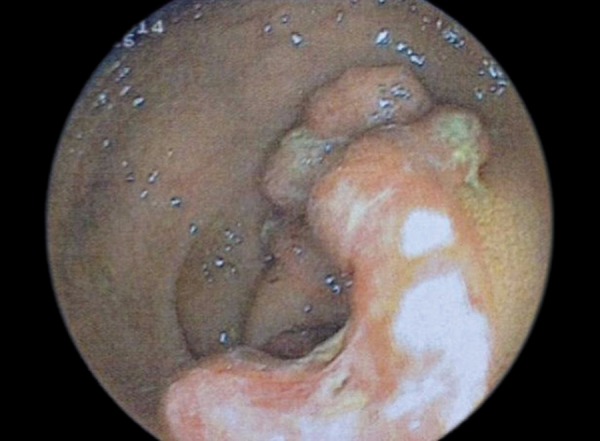
Tumoral mass in scirrhous feature detected on sigmoid colon

**Fig. 2:**
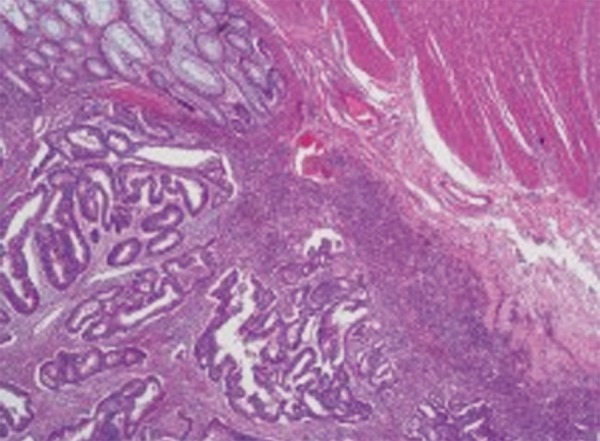
Pathological finding of the biopsy specimens obtained from the mass which was reported as colorectal carcinoma

**Fig. 3:**
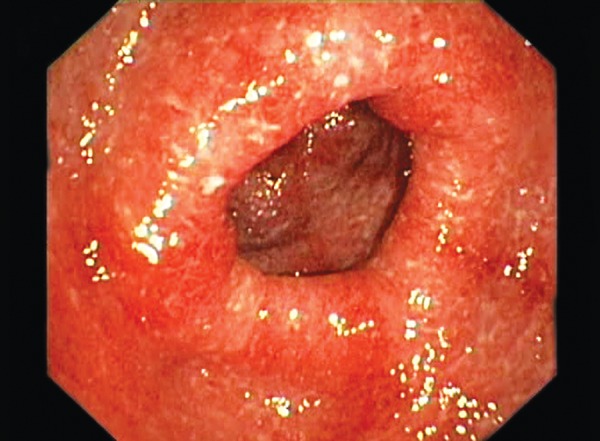
Hyperemic, edematous, spontaneous fragile and erosive mucosa of the colon

**Fig. 4:**
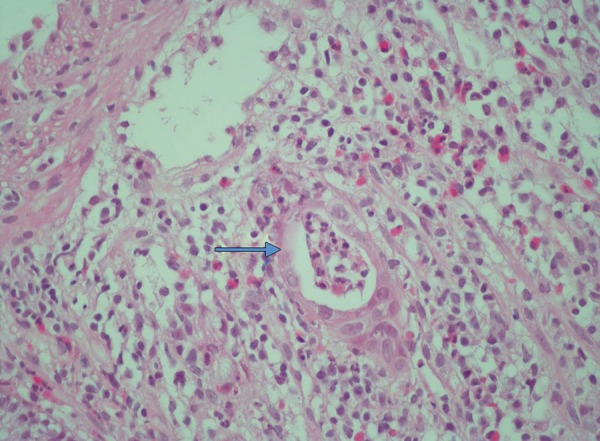
Chronic active colitis with distinct inflammation, moderate activation, crypt abscess, distortion and destruction

A 59-year-old female patient was admitted with history of abdominal pain for a year and rectal bleeding for a month. At colonoscopy, a tumor mass in scirrhous feature, narrowing the lumen and complicating the passage of the endoscope, was detected on sigmoid colon. Mucosa was normal through the last 10 cm of terminal ileum and the entire colon except tumoral tissue detected at sigmoid colon (Fig. 1). Pathological finding of the biopsy specimens obtained from the mass was reported as colorectal carcinoma (Fig. 2). At computed tomography (CT), asymmetric wall thickening on the rectosigmoid region, pollution of the adjacent mesenteric fat tissue and millimetric lymph nodes were observed. Low anterior resection was performed and pathological diagnosis was moderately differentiated adenocarcinoma with intact borders. Tumor was determined as T3N0M0. Oral capecitabine 3 gm/day for 14 days per month was administered. Two months later, she was admitted to the medical oncology clinic with complaint of a bloody diarrhea and bowel frequency of approximately 10 times/day. Fever and tachycardia was determined at physical examination. Other biochemical parameters were normal with erythrocyte sedimentation rate at 45 mm/hour. Stool examination revealed abundant leukocytes and erythrocytes with no parasitic cysts and ova. Oral capecitabine therapy was stopped. Intravenous ciprofloxacin and metronidazole treatments were administered. However, symptoms of the patients were not resolved on the seventh day of treatment (hemoglobin: 8.4 gm/dl, white blood cells: 5000/mm^3^, platelets: 283,000/mm^3^), colonoscopy was performed. On colonoscopy, although mucosa of the terminal ileum was normal, the mucosa of the cecum, ascending, transverse and descending colons and rectosigmoid region was hyperemic, edematous, spontaneous fragile and erosive (Fig. 3). The biopsy specimens obtained from the colonic mucosa was compatible with chronic active colitis with distinct inflammation, moderate activation, crypt abscess, distortion and destruction (Fig. 4). Diagnosis was considered as ulcerative pan colitis with severe clinical and endoscopic activation. Clinic remission was achieved with oral mesalazine 4 gm/day, mesalazine enema 4 gm/ day and metilprednisolone 32 mg/day. After induction of remission metilprednisolone was tapered and stopped. Oral mesalazine 4 gm/day and mesalazine enema 4 gm/ day were chosen for maintenance therapy. Colonoscopy 1 year after the low anterior resection revealed normal colo-nic mucosa with no recurrence of colorectal carcinoma.

Pathophysiology of the ulcerative colitis includes genetic susceptibility influenced by the luminal microbiota, which provides antigens and adjuvants that stimulate either pathogenic or protective immune responses. Environmental triggers as diet, the use of antibiotics and nonsteroid anti-inflammatory drugs, colonic ischemia stress or infection are necessary to initiate or reactivate disease.^2^

Takakura et al reported an ulcerative colitis case occurring 10 days after resection for sigmoid colon cancer and reviewed 8 other ulcerative colitis cases in the literature triggered by surgery for colorectal carcinoma. Interval for development of ulcerative colitis has been shown between 2 weeks and 8 years. While region that the colorectal carcinoma originates was sigmoid colon at 4 cases, rectum was the site of colorectal carcinoma at 4 cases. Surgical procedures were low anterior resection or sigmoidectomy. Chemotherapy background of the cases was indefinite.^3^

To date only two definitive reasons were given for a direct relationship between the colonic operation and the pathogenesis of ulcerative colitis. Takakura et al postulated that an ischemic condition induced by the operation might have caused the development of ulcerative colitis. In fact, it has been reported that the connection of microcirculation could be the etiology of ulcerative colitis, or strong emotional stress from the operation may be the cause as some reports suggest that psychological stress might lead to the pathogenesis, exacerbation, or relapse of ulcerative colitis, possibly triggering 40% of all cases of ulcerative colitis; Lim et al postulated that diversion colitis might have caused the development of ulcerative colitis as leukocytes were sensitized and activated in the vasculature of the inflamed diverted colon, circulated and recruited to the mucosa through the phenotypically similar vascular endothelium of the instream large intestine, finally causing an inflammatory process, which developed into ulcerative colitis.^3,4^

Another possibility is the presence of silent microscopic colitis as the etiological factor of colorectal carcinoma and triggering of manifest ulcerative colitis by perioperative factors. Katsanos et al reported a case of a silent ulcerative colitis adjacent to a regular sigmoid carcinoma. In his case at colonoscopy tumor mass was seen and the remaining colon mucosa was normal. Ulcerative colitis was detected on pathologic examination. This case may indicate that a normal looking mucosal ulcerative colitis increases the risk of colon cancer.^5^

Capecitabine is an orally administered prodrug of 5-fluorouracil commonly used in the treatment of advanced breast cancer, gastric cancer, colorectal cancer and esophageal cancer. Capecitabine induced colitis is rare with only four cases have been reported in the literature. The mechanism leading to colitis with ischemic features is unclear. Vasoconstriction induced colonic ischemia, mesenteric artery thrombosis due to injury to the endothelium and disruption of factors involved in fibrinolysis, direct mucosal injury and shift in intestinal micro flora which leads to activation of proinflammatory cytokines that results in mucosal necrosis may be the contributing factors.^6^

Except adjuvant chemotherapy regimen, the case we present; location of the tumor, interval for development of ulcerative colitis and surgical procedure for treatment of colorectal carcinoma were similar with the cases described in literature. Factors triggering ulcerative colitis after treatments for colorectal cancer might be the presence of silent microscopic colitis, capecitabine induced colonic ischemia and perioperative stress factors.

## CONCLUSION

Development of colitis after colon cancer may be associated with some causes as mutual genetic factors that take part at the pathophysiological mechanisms liable from occurrence of ulcerative colitis and colorectal carcinoma, chemotherapy agents, perioperative stress and underlying silent ulcerative colitis. It is unclear which role is certain. Increasing reports like this case will be useful resolving this issue.
